# From Experiments to Simulation: Shear-Induced Responses of Red Blood Cells to Different Oxygen Saturation Levels

**DOI:** 10.3389/fphys.2019.01559

**Published:** 2020-01-22

**Authors:** Elif Ugurel, Senol Piskin, Ali Cenk Aksu, Aysenur Eser, Ozlem Yalcin

**Affiliations:** ^1^Department of Physiology, School of Medicine, Koç University, Istanbul, Turkey; ^2^Research Center for Translational Medicine (KUTTAM), Koç University, Istanbul, Turkey; ^3^Department of Mechanical Engineering, Istinye University, Istanbul, Turkey; ^4^Department of Mechanical Engineering, The University of Texas at San Antonio, San Antonio, TX, United States; ^5^Graduate School of Biomedical Sciences and Engineering, Koç University, Istanbul, Turkey

**Keywords:** red blood cell, deformability, oxygenation, hypoxia, computational fluid dynamics, pulsatile blood flow

## Abstract

Red blood cells (RBC) carry and deliver oxygen (O_2_) to peripheral tissues through different microcirculatory regions where they are exposed to various levels of shear stress (SS). O_2_ affinity of hemoglobin (Hb) decreases as the blood enters the microcirculation. This phenomenon determines Hb interactions with RBC membrane proteins that can further regulate the structure of cytoskeleton and affect the mechanical properties of cells. The goal of this study is to evaluate shear-induced RBC deformability and simulate RBC dynamics in blood flow under oxygenated and deoxygenated conditions. Venous blood samples from healthy donors were oxygenated with ambient air or deoxygenated with 100% nitrogen gas for 10 min and immediately applied into an ektacytometer (LORRCA). RBC deformability was measured before and after the application of continuous 5 Pa SS for 300 s by LORRCA and recorded as elongation index (EI) values. A computational model was generated for the simulation of blood flow in a real carotid artery section. EI distribution throughout the artery and its relationships with velocity, pressure, wall SS and viscosity were determined by computational tools. RBC deformability significantly increased in deoxygenation compared to oxygenated state both before and after 5 Pa SS implementation (*p* < 0.0001). However, EI values after continuous SS were not significant at higher SS levels (>5.15 Pa) in deoxygenated condition. Simulation results revealed that the velocity gradient dominates the generation of SS and the shear thinning effect of blood has a minor effect on it. Distribution of EI was calculated during oxygenation/deoxygenation which is 5–10 times higher around the vessel wall compared to the center of the lumen for sections of the pulsatile flow profile. The extent of RBC deformability increases as RBCs approach to the vessel wall in a real 3D artery model and this increment is higher for deoxygenated condition compared to the oxygenated state. Hypoxia significantly increases shear-induced RBC deformability. RBCs could regulate their own mechanical properties in blood flow by increasing their deformability in hypoxic conditions. Computational tools can be applied for defining hypoxia-mediated RBC deformability changes to monitor blood flow in hypoxic tissues.

## Introduction

Red blood cells (RBC) play crucial roles in the circulatory system by carrying oxygen (O_2_) from respiratory surfaces to the tissues in demand. Most of the O_2_ is transported by hemoglobin (Hb) within RBCs. Diffusive transfer of O_2_ from RBC to the tissues is mainly determined by oxygen tension (Sprague et al., 2007). The blood becomes fully saturated with O_2_ at the high oxygen tension and Hb goes through a relaxed (R) state (oxyHb) that has a lower binding capacity for CO_2_, protons, chloride ions and organophosphates [2,3-diphosphoglycerate (DPG), ATP and inositol pentophosphate] (Stefanovic et al., 2013). As the blood enters the microcirculation, oxygen tension decreases that promotes O_2_ offloading from hemoglobin and a shift to a tense (T) structure (deoxyHb) (Jensen, 2009). DPG and ATP are at higher concentrations in RBC and have been shown to bind specifically to deoxyHb resulting in a marked lowering of its O_2_ affinity (Benesch et al., 1968; Nikinmaa, 1990). These organic phosphates are able to chelate Mg^2+^ ions. Upon deoxygenation, free intracellular Mg^2+^ levels are increased as DPG and ATP bind to deoxyHb and decreased on oxygenation (Bunn et al., 1971; Flatman, 1980). Physiological fluctuations in free intracellular Mg^2+^ have been shown to affect K/Cl cotransporter activity which is tightly controlled by phosphorylation reactions (Gibson et al., 1995, 2000; Bogdanova et al., 2009). Elevation of intracellular free Mg^2+^ also leads to Band3 phosphorylation, an anion exchanger and a link between membrane and underlying skeleton (Barbul et al., 1999). Oxygenation-deoxygenation transitions appear to evoke signaling cascades in RBC that modulate membrane ion transporters and the cytoskeleton by phosphorylation events which would further regulate mechanical properties of the cell membrane (Gibson et al., 2000; Barvitenko et al., 2005).

Red blood cells do not only function as passive O_2_ carriers, but they also act as a sensor of O_2_ requirements and adjust its O_2_ content to the level of O_2_ utilization within the physiological range of oxygen tension (pO_2_) (Ellsworth et al., 1995; Ellsworth, 2000). This would confer RBCs a central role in the process of matching local O_2_ supply with O_2_ demand, plausibly by regulating their own properties and functions (Sprague et al., 2007). RBCs can release ATP in a response to reduced O_2_ tension and mechanical deformation (Bergfeld and Forrester, 1992; Sprague et al., 1996). The amount of released ATP is also linked to a decrease in Hb O_2_ saturation (Jagger et al., 2001) and increasing amounts of ATP is accompanied by vasodilation (Dietrich et al., 2000). The primary mechanism for hypoxic vasodilation occurs via nitric oxide (NO) release from endothelial cells. However, several studies proposed a role for RBCs on the maintenance of NO bioavailability through hemoglobin deoxygenation that results in NO release from RBCs and subsequent NO-dependent vasodilation (Gladwin et al., 2004, 2006; Crawford et al., 2006; Grubina et al., 2007). NO rapidly interacts with hemoglobin molecule in RBCs (Azarov et al., 2005). When the blood is deoxygenated in the microcirculation, Hb switches to the T structure and this triggers the release of NO from SNO-Hb (Jia et al., 1996; Stamler et al., 1997). Conformational changes in Hb by oxygenation-deoxygenation cycles also modulate its binding affinity for RBC membrane proteins. The association between deoxyHb and Band3 promotes the dissociation of Band3-ankyrin bridge that leads to weakening of membrane-cytoskeletal interactions and changes in RBC membrane stability (Stefanovic et al., 2013; Chu et al., 2016). DeoxyHb also interacts with actin and tubulin, two of the main components of the cytoskeleton (Lebbar et al., 1987). Besides these associations between membrane proteins and Hb, the integrity of Glycophorin C-Protein 4.1 linkage was shown to be regulated by an oxygen-dependent way that deoxygenated cells retained less Glycophorin C in their membranes due to the reversible rupture of Protein 4.1 bridge upon deoxygenation (Chang and Low, 2001). Oxygen-mediated changes in the cytoskeleton could profoundly organize RBC properties that more likely by modulating RBC deformability which is a major determinant for proper blood flow (Viallat and Abkarian, 2014). The extreme deformability allows the RBCs to squeeze without any damage when passing through narrow capillaries in the microcirculation and this is mostly achieved by the viscoelastic properties of the cell membrane (Mohandas, 1992). Although oxygen-dependent regulations of RBC properties have been recognized for years, the data on the extent of cell deformability by oxygenation/deoxygenation is still controversial in literature (Hakim and Macek, 1988; Tuvia et al., 1992; Kaniewski et al., 1994; Parthasarathi and Lipowsky, 1999; Wei et al., 2016). Earlier studies have asserted that RBCs became less deformable in hypoxic conditions (Hakim and Macek, 1988; Tuvia et al., 1992; Kaniewski et al., 1994; Parthasarathi and Lipowsky, 1999), however, a current study revealed elongation of RBCs in response to shear stress increased as oxygen tension decreased (Wei et al., 2016).

This study evaluated shear-induced RBC deformability by an ektacytometer under oxygenated and deoxygenated conditions and performed a realistic simulation of RBC dynamics in blood flow by computational fluid mechanics. In the experimental part of the study, RBC deformability was measured under fluid shear stresses by the means of elongation index (EI) of RBCs at different oxygen saturation levels. In the computational part of the study we aim to model the blood flow simulation in a realistic carotid artery to predict the distribution of the EI with regard to the shear stress and the oxygen saturation level. Although this is a relatively large artery compared to the arterioles or capillaries, the main idea is to predict the distribution of EI in a patient specific artery. Indeed, this information could be useful in terms of diagnosis and treatment of several diseases. For instance, studies reported that children with sickle cell anemia have a high incidence of strokes, and transcranial doppler (TCD) identifies at-risk patients by measuring blood velocities in large intracerebral arteries (Rivera et al., 2016). Oxygen concentration in large arteries can also be a clear understanding of the systemic distribution of the oxygen and it should be helpful for assessing the status of the human body and for offering valuable guidance for the prevention and intervention of diseases (Yan et al., 2018). Furthermore, the velocity thus shear rate distribution is expected to be very similar to large arteries unless the continuum mechanics is not valid: relatively higher close to the wall and stagnation points and lower at the center of the artery. Taken together, the aim of the simulation in the present study is to obtain the distribution of RBC EI throughout the blood circulation at different oxygen saturation levels. Besides the EI, other hemodynamic parameters such as wall shear stress and pressure were quantified for their possible collaboration among each other.

## Materials and Methods

### Blood Sampling and Preparation

Venous blood samples were collected from antecubital veins of healthy donors (*n* = 10) into sodium-heparinized vacutainers (BD Vacutainer, 15 IU/ml). An informed written consent was obtained before the sampling. The use of human blood was in accordance with The Code of Ethics of the World Medical Association (Declaration of Helsinki). The study was approved by The Ethics Committee of Koc University School of Medicine (IRB: 020/2012). All participants were adult male volunteers in the 25–50 age range. The hematocrit value of blood samples was measured by capillary tubes using microcentrifuge and adjusted to 0.4 l/l with autologous plasma. All experiments were carried out within 4–6 h after blood collection.

### Preparation of Resealed Ghosts

Red blood cells were collected from venous blood samples by centrifugation at 2500 × *g* for 5 min. Plasma and buffy coat were removed from pelleted RBCs. Ghosts were prepared from RBCs as described previously (Manno et al., 2002; Aryee and Takakuwa, 2013). Briefly, RBCs were washed three times with 10 mM Tris–HCl buffer (pH 7.4) containing 120 mM KCl. Cells were lysed and washed with 35 volume of 5T5K buffer (5 mM Tris–HCl, 5 mM KCl, pH 7.4) supplemented with 1 mM MgCl and 10 mM ATP. Ghosts were washed at 18,000 × g until supernatant appeared free from hemoglobin and they became pink-white. A small volume of concentrated buffer (1:10) containing 150 mM KCl, 1 mM MgCl2 and 1 mM dithiothreitol was added to ghost suspension to restore isotonicity. RBC ghosts were resealed at 37°C for 40 min before deformability measurements.

### Experimental Design for Oxygenation/Deoxygenation Process

Whole blood and RBC ghost samples were diluted with polyvinylpyrrolidone (PVP) solution (29.8 mPa.s, 304 mOsm/kg, pH 7.4, Mechatronics, Hoorn, Netherlands) in polypropylene tubes with a dilution ratio of 1:200. PVP solution contains no ions or ionic compounds to affect cell properties or oxygenation state of Hb molecule. Samples were either oxygenated with ambient air with an air pump or deoxygenated with 100% nitrogen gas from a gas cylinder with 10 psi pressure for 10 min at room temperature. Both air and nitrogen gas were pre-wetted by bubbling through distilled water prior to oxygenating/deoxygenating the samples and this process continued during the measurements. Oxygenated or deoxygenated sample in PVP was immediately applied to the measuring chamber of the device (LORRCA OxygenScan, Mechatronics, Netherlands) which was connected to the gas source. Ambient air or nitrogen gas was continuously supplied into the device as the shearing proceeded. Oxygen saturation of samples was measured by a fiber optic oxygen transmitter (Fibox 4, PreSens – Precision Sensing GmbH, Germany) with sensor probes located in the rotating cylinder of the device. Oxygen saturation in the sample was monitored throughout the experiments. The measurements of deformability were taken only when the oxygen saturation was between 20–22% in the oxygenated sample and between 0.5–3% in the deoxygenated sample.

### Application of Shear Stress to Blood and RBC Ghost Samples

A laser-assisted optical rotational cell analyzer (LORRCA OxygenScan, Mechatronics, Netherlands) was used to apply continuous shear stress (SS) to blood and ghost samples for 300 s. The level of applied SS was at 5 Pa which is physiologically relevant (Brands et al., 1995; Malek et al., 1999). LORRCA has a Couette type shearing system with static and rotating cylinders made of glass. The sample is applied into the gap (measuring chamber) between the two cylinders. The shearing force is driven by the rotating cylinder and the viscosity of the suspending medium (PVP, viscosity: 29.8 mPa.s). A diode laser is integrated in the static cylinder and the laser beam is diffracted by RBC as it traverses through the blood sample. The diffraction pattern is monitored by a CCD camera and then analyzed by an integrated computer. The measurements of RBC deformability were recorded as EI and it was calculated as follows: EI = (*a* − *b*)/(*a* + *b*) where “a” is the vertical axis and “b” is the horizontal axis of the diffraction pattern. The experiments were performed at 37°C.

### Assessment of Deformability

Deformability of RBCs and RBC ghosts was determined by the change in the EI within the applied SS range. Deformability was measured by applying ten different SS as follows: 0.3, 0.53, 0.94, 1.65, 2.91, 5.15, 9.09, 16.04, 28.32, and 50 Pa. Then, a constant SS of 5 Pa was applied continuously for 300 s and deformability was measured again with a maximum delay of 10 s under same conditions. Maximal RBC elongation index (EI_max_) and the SS required for one-half of this maximal deformation (SS_1__/__2_) were calculated by the linear Lineweaver–Burke model (Baskurt et al., 2009). The SS_1__/__2_/EI_max_ ratio was also calculated as a normalized measure of SS_1__/__2_ (Baskurt and Meiselman, 2013). Deformability of RBCs and RBC ghosts was evaluated before and after the application of continuous SS.

### Simulation of Blood Flow in the Carotid Artery

Three-dimensional carotid artery bifurcation geometry was taken from GrabCAD community^1^ (Supplementary Figure 1). It has been generated from a luminal casting. The diameters of the inlet (main carotid artery), outlet 1 (internal carotid artery) and outlet 2 (external carotid artery) are 6.3, 4.5, and 3 mm, respectively. A commercial Computational Fluid Dynamics (CFD) solver, FLUENT 17.0 (Ansys, Inc., Canonsburg, PA, United States) was adopted for this study. The CFD software was configured to implement a multi-grid artificial compressibility solver for incompressible non-Newtonian flows and employs a second-order accurate numerical discretization scheme. The grid generator of Ansys Mesher 17.0 (Ansys, Inc., Canonsburg, PA, United States) was employed to produce 3D tetrahedral mesh elements. Assessment of the mesh quality was defined as devoid of high cell skewness, as there is a known relationship between mesh quality, simulation convergence and convergence time (Antiga et al., 2002). Also, a mesh sensitivity analysis has been performed to find the optimum mesh element size as shown in Supplementary Figure 2.

### Governing Equations for the Simulation of Blood Flow

The blood flow in the carotid artery is assumed to be incompressible and laminar. The governing equations of the model are the continuity and the Navier–Stokes equations and can be stated as follows:

∇⋅u=0,

$$\text{ρ}\,\frac{\partial u}{\partial t}+\text{ρ}\,\nabla \cdot u\,u=-\nabla p+\nabla \cdot \text{μ}\,\left(\nabla u+\nabla^{T}u\right)+\text{ρ}\,g,$$

where μ, ρ, g, *p*, and *u* denote the viscosity and the density of the fluid, the gravitational acceleration, the pressure, and the velocity vector respectively. Since blood is a non-Newtonian fluid, μ is not constant due to shear thinning material property. In this study, the viscosity is considered as a function of shear rate and hematocrit ratio. To represent this dependency, the Casson model was used here (Perktold et al., 1991). The model can be written as:

$$\text{μ}=\left\{ \begin{array}{c} \frac{{\text{μ}}_{\infty }^{2}}{\dot{\text{γ}}}+\frac{2\,{\text{μ}}_{\infty }\,N_{\infty }}{\sqrt{\dot{\text{γ}}}}+N_{\infty }^{2},\dot{\text{γ}}>0,\\ 0.0035,o\,t\,h\,e\,r\,w\,i\,s\,e. \end{array}\right.$$

Here, $\dot{\text{γ}}$ is the shear rate, μ_0_ the plasma viscosity, ${\text{μ}}_{\infty }^{2}$ the Casson yield stress, and $N_{\infty }^{2}$ the Casson viscosity. Shear rate is defined as $\dot{\text{γ}}=2\,\sqrt{\left(D:D\right)}$ where D=12(∇u+∇Tu). The *N*_∞_ and μ_∞_ are expressed as the functions of the hematocrit ratio (Hct) and given by

$$N_{\infty }=\sqrt{{\text{μ}}_{0}\,{\left(1-\text{Hct}\right)}^{-0.25}}$$

and

$${\text{μ}}_{\infty }=\sqrt{\left(0.625\cdot \text{Hct}\right)}$$

### Boundary Conditions for the Carotid Artery Model

**FIGURE 1:**
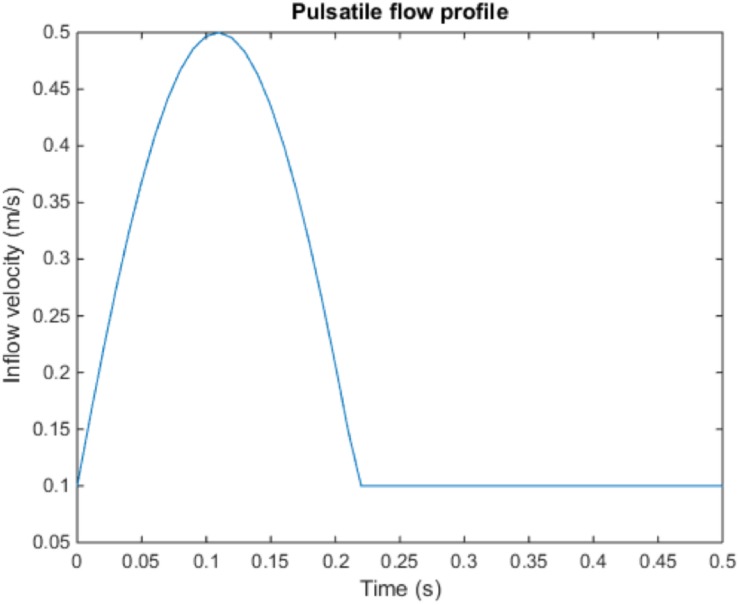
Pulsatile flow profile used as an inlet boundary condition of the carotid artery model. The pulse has a period of 0.5 s and a frequency of 120 beats per minute.

Pulsatile blood flow is applied in the simulations by imposing a time-varying velocity boundary condition at the inlet. A physiological pulse is approximated as a sinusoidal profile as shown in Figure 1. The period of the pulse is 0.5 s (i.e., frequency of 120 beats per minute) with minimum (diastolic) and maximum (systolic) velocities set as 0.1 and 0.5 m/s, respectively. The current model was proposed by Sinnott et al. (2006). The static gauge pressure at both outlets is set as 13.332 kPa which is the average pressure of the systolic and diastolic pressures of the healthy human. Wall boundaries are assumed to be rigid. A time step size test has been performed to find the maximum time step size necessary for the required accuracy as presented in the Supplementary Figure 2.

### Statistical Analysis

Data are presented as mean ± standard error (SE) unless otherwise stated. Two-way mixed model ANOVA was performed for the comparisons of EI-SS data in oxygenated and deoxygenated states before and after continuous 5 Pa SS. Bonferroni’s multiple comparisons test was performed as a *post hoc* test and significance level was defined as *p* < 0.05. SS_1__/__2_ and EI_max_ values were calculated by Lineweaver-Burke approach. All data analyses were performed with GraphPad v6 software package (GraphPad software, La Jolla, CA, United States).

## Results

### SS-Induced Improvements of Deformability in the Oxygenated and Deoxygenated States

**FIGURE 2:**
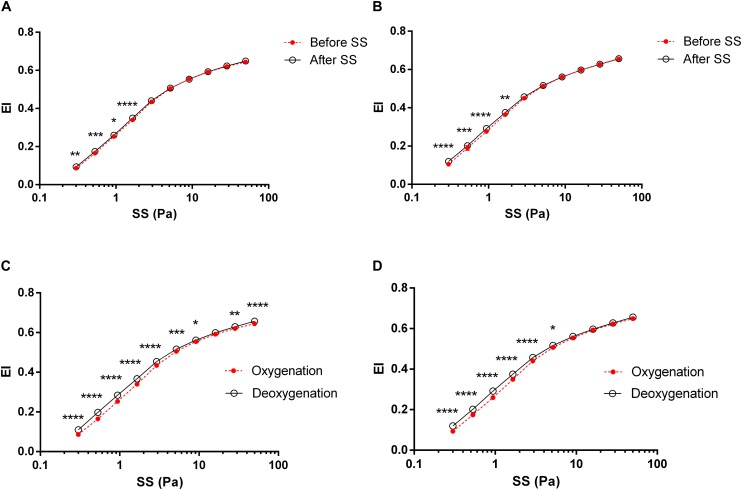
RBC deformability curves obtained by the change in elongation index (EI) versus shear stress (SS). Each circle represents an EI value corresponding to a different SS level (0.3, 0.53, 0.94, 1.65, 2.91, 5.15, 9.09, 16.04, 28.32, and 50 Pa). Data is collected from 10 different subjects with two replicative measurements and presented as mean ± standard error (SE). **(A)** The change in EI before and after 5 Pa SS in oxygenated conditions, **(B)** The change in EI before and after 5 Pa SS in deoxygenated conditions, **(C)** EI difference between oxygenation and deoxygenation before 5 Pa SS, **(D)** EI difference between oxygenation and deoxygenation after 5 Pa SS. ^****^p < 0.0001, ^∗∗∗^*p* < 0.001, ^∗∗^*p* < 0.01, ^∗^*p* < 0.05.

The application of a continuous SS to blood at 5 Pa improved RBC deformability that EI values significantly increased at low SS levels between 0.3–1.65 Pa after 5 Pa SS application (Figures 2A,B, *p* < 0.05, *p* < 0.01, *p* < 0.001 or *p* < 0.0001). These SS-induced improvements of deformability were recorded in both oxygenated and deoxygenated conditions. Before applying 5 Pa SS, RBC deformability significantly increased in deoxygenated blood compared to oxygenated samples at all SS levels except 16.04 Pa (Figure 2C, *p* < 0.05, *p* < 0.01, *p* < 0.001 or *p* < 0.0001). After continuous 5 Pa SS, a substantial increase was recorded between 0.3–5.15 Pa range considered as low SS levels (Figure 2D, *p* < 0.05 or *p* < 0.0001). RBC deformability significantly differed between oxygenated and deoxygenated conditions and the difference was more profound before 5 Pa SS (Figure 2C).

**FIGURE 3:**
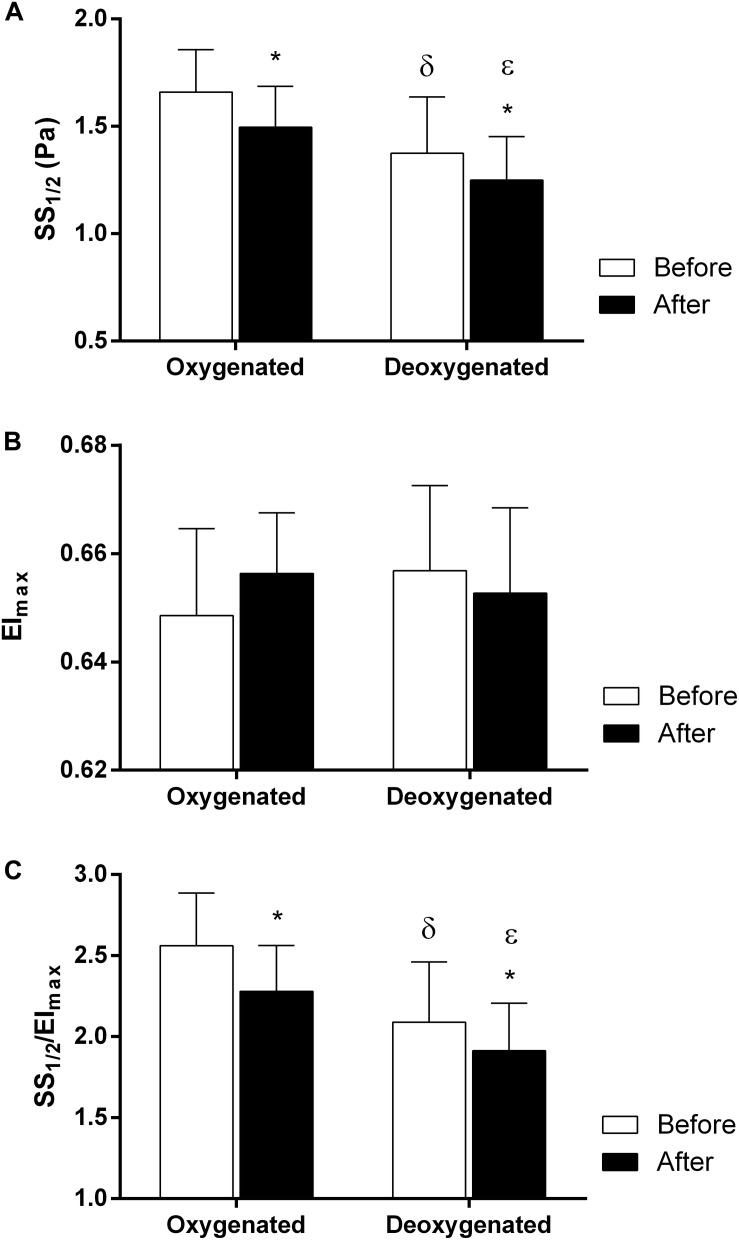
Maximal elongation and half shear stress parameters. Data is collected from 10 different subjects with two replicative measurements and presented as mean ± standard deviation (SD). SS_1__/__2_ **(A)**, EI_max_ **(B)**, SS_1__/__2_/EI_max_ **(C)** parameters in oxygenated and deoxygenated conditions before and after 5 Pa SS application. ^∗^*p* < 0.05 (The difference between before and after SS), δ*p* < 0.001 (The difference in SS_1__/__2_ values between oxygenated and deoxygenated conditions), ε*p* < 0.0001 (The difference in SS_1__/__2_/EI_max_ values between oxygenated and deoxygenated conditions before SS).

The maximal RBC elongation (EI_max_) and SS level required to reach one half of this maximal elongation (SS_1__/__2_) were calculated from deformability curves. SS_1__/__2_ values were significantly reduced after 5 Pa SS in both oxygenated (*p* < 0.01) and deoxygenated (*p* < 0.05) conditions (Figure 3A). Deoxygenation significantly decreased SS_1__/__2_ values before and after SS application compared to the oxygenated state (Figure 3A, *p* < 0.001). EI_max_ values were not changed by continuous SS and not differed between oxygenation and deoxygenation (Figure 3B). SS_1__/__2_/EI_max_ values, the normalized ratio of SS_1__/__2_, were significantly reduced after SS application in both conditions (Figure 3C, *p* < 0.01 for oxygenation and *p* < 0.05 for deoxygenation). This ratio was significantly decreased in deoxygenated blood compared to oxygenated samples both before (*p* < 0.0001) and after (*p* < 0.001) SS application (Figure 3C).

Elongation index values of RBC ghosts were deteriorated at high SS levels (>5.15 Pa, data not shown). Continuous 5 Pa SS application did not improve ghost deformability. No significant changes were recorded in the deformability of RBC ghosts between oxygenated and deoxygenated states (Supplementary Table 2).

### *In silico* Simulation of Experimental Results: Derivation of Analytical Function for Elongation Index

*In silico* blood flow simulation was performed by a realistic carotid artery geometry. Inputs of the model are the results of the measurements regarding the EI. The calculated EI values for each applied shear stress are shown in Supplementary Table 3 for both oxygenated and deoxygenated blood samples. Shear stress applied varies between 0.3 and 50 Pa. The corresponding EI changes from 0.084 to 0.643 for oxygenated and 0.111 to 0.658 for deoxygenated samples. As the table presents, deoxygenated samples have always higher elongation.

The SS versus EI are provided in a discrete form for 10 different values in Supplementary Table 3. During the simulation, there would be shear stress values other than the available in the table due to the continuum character of the physiology. Thus, two analytical formulations, which represent the relationship between SS and EI, are required. These relationships are obtained by fitting the discrete data to an exponential model using non-linear least squares curve fitting method. The two-element exponential function obtained for oxygenated blood samples is shown in Eq. 1:

(1) $$\text{EI}=0.5464\cdot {\text{e}}^{0.00356\cdot \text{SS}}-0.5224\cdot {\text{e}}^{-0.5395\cdot \text{SS}}$$

**FIGURE 4:**
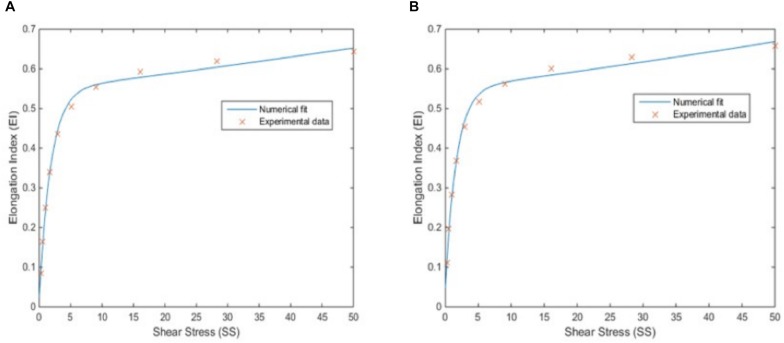
Correlation between elongation index (EI) and shear stress (SS). EI-SS correlation for oxygenated blood samples with a 2-element exponential fit, for which *R*^2^ is 0.9952 (*p* < 0.05) **(A)** and for deoxygenated blood samples with a 2-element exponential fit, for which *R*^2^ is 0.9940 (*p* < 0.05) **(B)**.

Here, *R*^2^ = 0.9952 for the model and (*p* < 0.05) for the coefficients of the model. Figure 4A shows the plot of the fitted function versus the experimental data. The same procedure is repeated for deoxygenated blood sample data and the fitted function is found as in Eq. 2:

(2) $$\text{EI}=0.5483\cdot {\text{e}}^{0.003966\cdot \text{SS}}-0.5006\cdot {\text{e}}^{-0.598\cdot \text{SS}}$$

Here, *R*^2^ = 0.9940 for the model and (*p* < 0.05) for the coefficients of the model. Figure 4B shows the plot of the fitted function versus the experimental data. Both Figures 4A,B and high *R*^2^ values show a good match between discrete data and analytical function which is required for an accurate simulation model.

### Velocity Distribution and Streamlines

**FIGURE 5:**
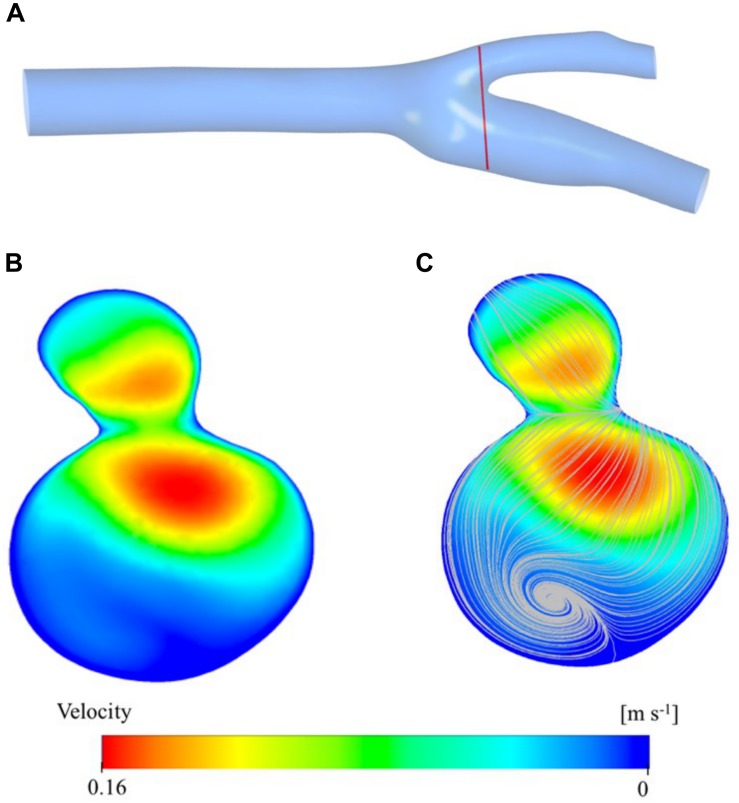
Velocity distribution at a cross section close to the bifurcation region is presented here: the location of the velocity magnitude contours **(A)**, vorticity streamlines **(B)**, and cross-sectional plane **(C)**.

Figure 5 shows the velocity contour plot at a selected region of the carotid artery model. The region is very close to the bifurcation of the main carotid artery into the internal and external carotid arteries (Figure 5A). This result is used mainly obtained for validation of the simulation study. As expected, the main artery flow is divided into daughter arteries and the maxima of the velocity profile are diverted from the midpoint of the cross-section of the larger vessel to the side regions of the smaller vessels (Figure 5B). While the converging walls of the bifurcation are closer to the higher velocity zones, diverging walls have relatively low-velocity zones. Especially, the internal artery has a large stationary zone which causes secondary (rotational) flow and thus vorticity. Vorticity streamlines are visualized in Figure 5C and clearly show the circulation of the blood in the sinus of the internal artery. Those types of zones are prone to be high blood residence time regions and correlated with plaque deposition.

**FIGURE 6:**
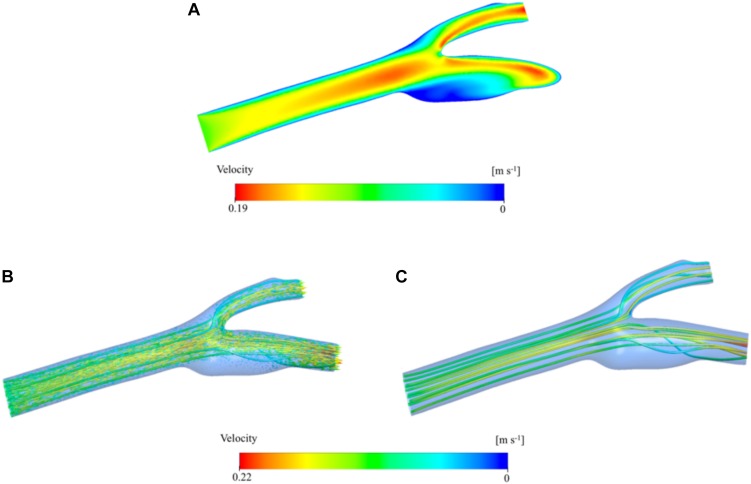
Velocity distribution color-coded by velocity magnitude. Contours are shown in the two-dimensional midplane in the axial direction **(A)**. Velocity vector distribution in the three-dimensional artery geometry. The vectors are colored by the velocity magnitude and the vector size is scaled based on the velocity magnitude **(B)**. Velocity streamlines are presented inside the 3D domain and colored by velocity magnitude **(C)**.

Results from the bifurcation region can further be verified by visualizing the velocity on the mid-axial plane and the streamlines on the whole 3D domain of the artery. Figure 6A presents a contour plot of the velocity distribution at the midplane along the longitudinal axis of the main carotid artery. Here, we can clearly see the developed flow at the end of the main carotid artery and bifurcation of the flow between internal and external arteries. Just after the bifurcation region, flow profile maxima are close to the converging walls of the vessel, but the flow redevelops before reaching the outlets and the maxima come to the center again. These observations are supported by visualization of 3D velocity vectors (Figure 6B) and streamlines (Figure 6C) color-coded by velocity magnitude. Size of the vectors is scaled based on the velocity magnitude. Secondary flow within the sinus zone of the internal artery can be recognized by small velocity vectors not aligned with the primary flow direction (i.e., streamlines). Stagnation point at the bifurcation region is also visible by relatively very low velocity (Figure 6A) and no streamlines at the corresponding location (Figures 6B,C).

### Pressure and Wall Shear Stress Distribution

**FIGURE 7:**
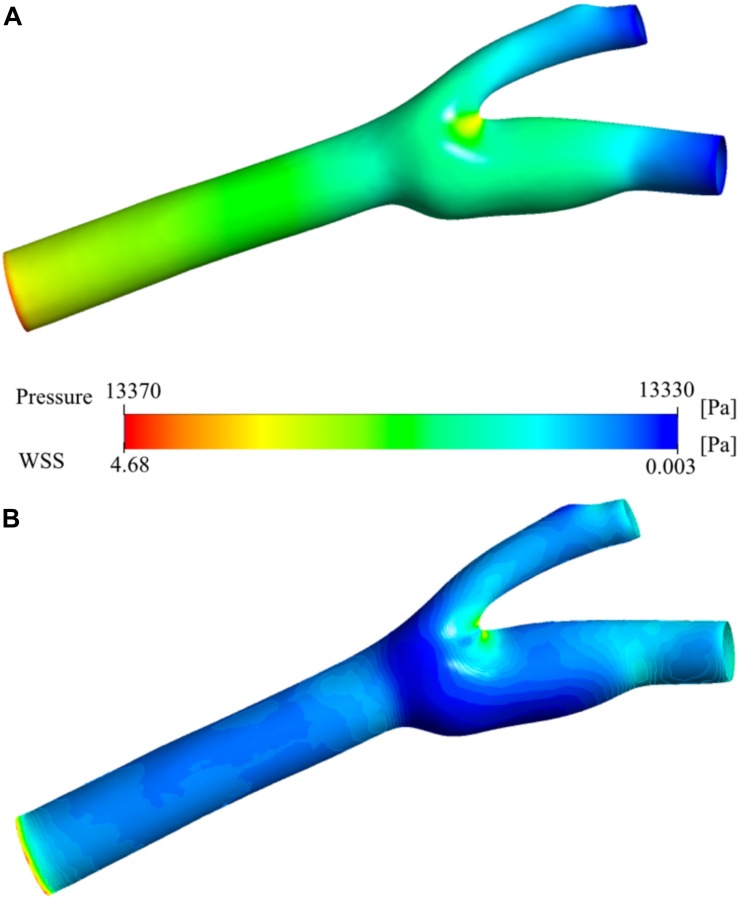
Pressure and wall shear stress. Pressure distribution **(A)** and wall shear stress (WSS) **(B)** on the carotid artery wall.

Figure 7A shows the pressure distribution on the carotid artery wall. There is a smooth gradient from the main carotid artery inlet to the internal and external outlets as expected from laminar blood flow. The stagnation point at the bifurcation region is visible as the pressure increases at the low-velocity zone. Figure 7B presents the wall shear stress (WSS) distribution. High WSS zones are visible especially around the bifurcation region and narrowing regions (stenosis) of both internal and external carotid arteries. These regions have relatively low (stagnation point) and high (stenosis) velocity distributions (see Figure 6). The main reason for high WSS at those regions is the high-velocity gradient zones which cause high shear rates. The sinus region has relatively low WSS due to very low velocity, vorticity, and steady flow. High blood residence time is also correlated with this behavior.

### Viscosity Distribution

**FIGURE 8:**
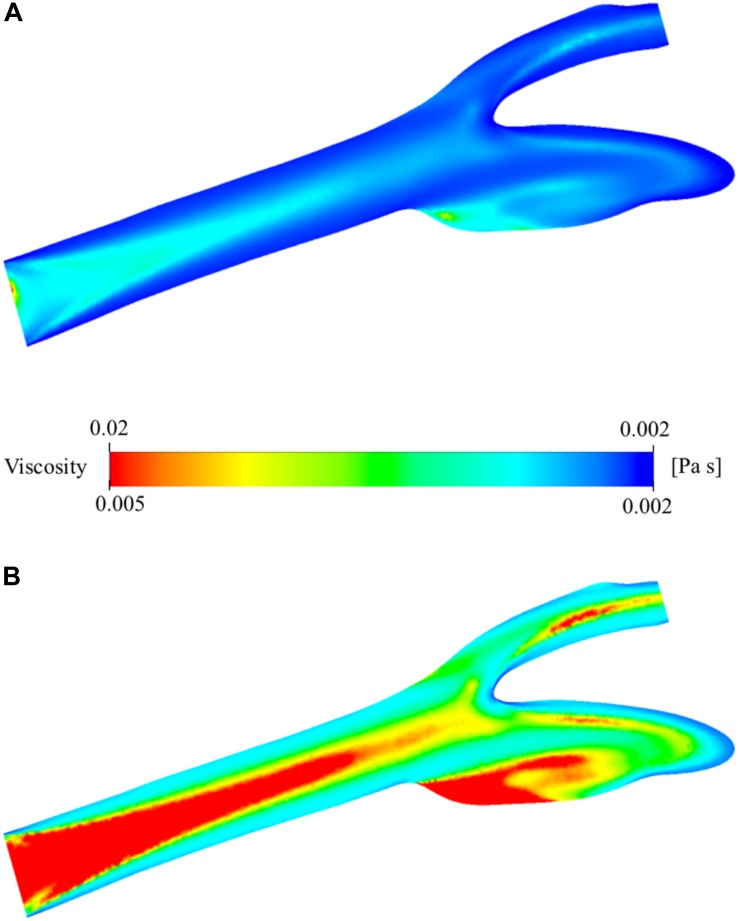
Viscosity distribution on the two-dimensional midplane in the axial direction. **(A)** shows the range with minimum and maximum viscosity values while **(B)** shows the scaled-down range of the viscosity for detailed visualization.

The viscosity of the blood varies by space and time during the simulation due to the change of shear rate. The relationship between viscosity and shear rate is constructed by the non-Newtonian Casson model. The spatial distribution of the viscosity is presented in Figure 8A. It has a minimum of 1.886 × 10^–3^ Pa⋅s and a maximum of 2.072 × 10^–2^ Pa⋅s. They are largely different from the nominal blood viscosity: 3.500 × 10^–3^ Pa⋅s. To analyze the distribution of viscosity better, the range of the legend has been scaled down so that it will be between 1.886 × 10^–3^ and 5.000 × 10^–3^ Pa⋅s instead of 1.886 × 10^–3^ and 2.072 × 10^–2^ Pa⋅s (Figure 8B). The middle section of the main carotid artery starts with a relatively high viscosity value and as the flow develops, the high viscosity region gets narrower. The high viscosity regions are generally far from the artery walls due to the shear thinning characteristics of the non-Newtonian model. The viscosity tends to increase with decreasing shear rate. There is also a large zone with high viscosity, at the internal carotid artery sinus region. This is due to the stationary flow zone and thus again very low shear rate.

### Elongation Distribution for the Simulation of Oxygenated and Deoxygenated Blood Flow

**FIGURE 9:**
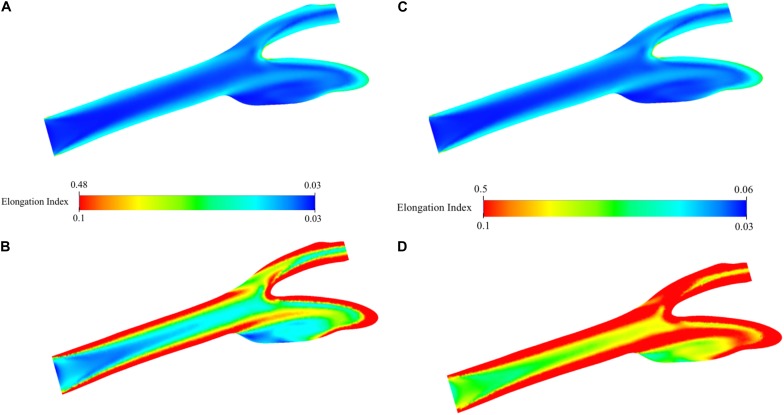
Elongation index distribution for blood flow simulation. Contours are shown on the two-dimensional midplane in the axial direction. **(A)** shows the range with minimum and maximum EI values while **(B)** shows the scaled-down range of EI values for detailed visualization for oxygenated blood. **(C)** shows the range with minimum and maximum EI values while **(D)** shows the scaled-down range of EI values for detailed visualization for deoxygenated blood.

Distribution of EI in the carotid artery (axial mid-plane) for the oxygenated blood during physiological flow is presented in Figure 9A. The figure shows that the highly elongated cells are concatenated especially close to the artery walls due to high-velocity gradient and thus shear rate. The highest EI is observed around the stagnation region due to zero velocity and thus highest shear rate. This EI is 0.480 and between 0.435 and 0.506 which are the experimentally measured values at the corresponding shear stress levels between 2.91 and 5.15 Pa (Supplementary Table 3). The minimum value is 0.033 and actually lower than the lowest EI value measured by deformability test. To better observe the distribution of EI, the range of the legend has been scaled down so that it will be between 0.033 and 0.100 instead of 0.033 and 0.481 (Figure 9B). This figure clearly shows the high EI values around the main carotid artery walls. After the bifurcation, this behavior changes and converging walls of internal and external walls have higher EI values compared to diverging walls. As the flow develops again, the diverging walls start to have high EI values as well. A visual map of EI (Figure 9B) is almost a negative image of the corresponding viscosity map (Figure 8B). Similar analysis has been performed for the simulation of deoxygenated blood flow (Figures 9C,D). Both minimum and maximum values of the EI are higher than that of the oxygenated blood flow simulation. This is expected since higher EI values have been observed for deoxygenated blood samples in deformability measurements. To compare with the oxygenated blood flow simulation, the range of the legend has been scaled down so that it will be between 0.033 and 0.100 instead of 0.052 and 0.500 (Figure 9D). Comparison of Figures 9B,D suggests that the EI at each point of the artery is higher for deoxygenated than the oxygenated blood samples.

## Discussion

This study demonstrated that deoxygenation substantially improves RBC deformability and these improvements are also present after shear exposure. The computational models in the study revealed that the extent of RBC deformability increases as RBCs approach to the vessel wall in both oxygenated/deoxygenated conditions and this increment is higher in the deoxygenated state than the oxygenated condition.

Red blood cell deformability refers to the ability of cells to adapt their shapes to dynamically changing flow conditions to minimize their resistance to flow. RBCs respond to applied shear forces in blood flow by their capacity to deform which is particularly important for the passage of RBCs through microcirculation (Baskurt and Meiselman, 2003). RBC deformability is known to increase progressively during the application of physiological levels of shear stress (SS) (Meram et al., 2013). The evaluation of shear-induced RBC deformability is fundamental to our understanding of RBC mechanical behavior in the microcirculation where they are exposed to various SS levels. In this study, we investigated the effects of oxygenation and deoxygenation on RBC mechanical responses by application of 5 Pa SS which is physiologically relevant. We demonstrated that deoxygenation significantly increased RBC deformability both before and after SS application that could be expressed as oxygenated/deoxygenated state alters shear-induced responses of RBCs. An elevation in EI values by deoxygenation was significant almost at all SS levels before 5 Pa application while it was only significant at low SS levels (0.3–5.15 Pa) after 5 Pa exposure (Figures 2C,D). The improving effect of deoxygenation on RBC deformability was also confirmed by parameterization of EI-SS curves as it was manifested with lower SS_1__/__2_ and SS_1__/__2_/EI_max_ values in deoxygenated blood than the oxygenated blood samples (Figure 3).

The experimental results of the study are supported by *in silico* simulation of the blood flow using the data of deformability measurements. This part of the study is a research study to test the capability of computational tools to quantify the effect of hemodynamic factors on human physiology. The physical tests performed in the current study are generally limited by physical measuring capacity of the application setup and imaging tools. Here, deformability measurements were performed between 0.3 and 50 Pa (Supplementary Table 1). But the actual values are beyond these limits as revealed by the simulation results (Figure 9). Experimental results provide quantitative data for a number of discrete data but the total *in vivo* effect on the artery system needs more advanced tools. CFD can be a good subsidiary for this purpose. It can quantify the effect in a 3D artery or even the whole arterial system and capillaries. Furthermore, the realistic geometry sheds light on each region of the arterial system which is not predictable easily if not impossible by the proposed experimental setup. For instance, the simulation results suggest that the sinus region of the internal carotid artery wall have relatively low EI value although other walls have higher EI value distribution. Also, the stagnation point of the bifurcation from the main carotid artery into internal and external arteries has the highest EI value both for oxygenated and deoxygenated blood. The findings of the simulations have been cross-checked using several parameters such as pressure, velocity, vorticity and viscosity distribution through the carotid artery cross-sectional planes. The viscosity distribution (Figure 8) has been verified by velocity distribution (Figure 6): higher velocity gradients cause lower viscosity due to non-Newtonian characteristics of the blood (shear thinning effect). The EI distribution is almost the negative image of the viscosity distribution (Figures 8, 9). This is due to the higher shear causes higher EI and lower viscosity. The EI distribution of deoxygenated blood has higher values in each region of the artery compared to oxygenated blood. For both conditions, the highly elongated cells are concatenated close to the wall except the sinus region.

Contribution of oxygenation/deoxygenation to RBC deformability has been controversial in literature throughout the years. To our knowledge, La Celle and Weed were the first authors reported that low oxygen pressure is a cause of RBC membrane rigidity (La Celle and Weed, 1970). Some studies supported this finding of La Celle and Weed (Tuvia et al., 1992; Barbul et al., 1999; Parthasarathi and Lipowsky, 1999; Uyuklu et al., 2009a, b) while others did not observe a significant change in RBC deformability between oxygenated and deoxygenated conditions (Kaniewski et al., 1994; Chang and Low, 2001). The main reason for this discrepancy may lie in the methodology used to measure the deformability. In a study by Tuvia et al. (1992), an optical method was used to measure the membrane fluctuations whereas in another study by Parthasarathi and Lipowsky (1999) the transit time of fluorescently labeled RBCs was measured. Another reason could be the experimental setup that is hard to maintain partial oxygen or nitrogen pressure in the environment and the extent of oxygen saturation in the blood. An *in vivo* study on human subjects reported that RBC deformability was reduced in mild hypoxia (12–16% O_2_) and increased under severe hypoxia (<10% O_2_) (Grau et al., 2016). Nevertheless, a recent study by Wei et al. (2016) revealed that RBC velocity and deformability increases as pO_2_ gradually decreases in a microfluidic channel. This finding indicates that brief deoxygenation i.e., in microseconds, can affect the mechanical properties of RBCs (Wei et al., 2016). In our study, we confirmed the findings of Wei et al. (2016) and demonstrated that shear-induced RBC deformability significantly increases by longer term (10 min) deoxygenation compared to the normal oxygenated state. This may be explained as RBCs regulate their own properties by increasing their deformability in hypoxic conditions in order to pass easily through the capillaries where they are exposed to various levels of shear forces. In addition, we showed that RBC deformability is improved by physiological shear stress both in normoxic and hypoxic conditions. Shear stress at physiological levels was previously shown to improve RBC deformability in normoxia, presumably for the maintenance of adequate microcirculatory perfusion (Meram et al., 2013). In this study, we demonstrated hypoxia also improves RBC deformability when the oxygen saturation was 0.5–3% in the blood. We expected to see more significant improvements in deformability as RBCs are exposed to both shear stress and hypoxia. However, deoxygenation for 10 min together with continuous shear stress application for 5 min reduced hypoxia-induced improvements of deformability where no significant difference was recorded between the oxygenated and deoxygenated state at high SS levels (>5.15 Pa). This could be due to the depletion of intracellular energy stores by long term hypoxia (10 min) during which RBCs may become less tolerant to shear stress and subsequently more sensitive to mechanical damage. Indeed, the attenuation of ðÎ_2_ leads to ATP release from human RBCs (Bergfeld and Forrester, 1992; Ellsworth et al., 1995). On the other hand, most cells have the capability of adaptation to hypoxia by decreasing their energy-consuming processes (Michiels, 2004). This might explain the reductions in hypoxia-induced improvements of deformability due to shear stress application although deoxygenation could alone increase RBC deformability. In addition, shear-induced responses of RBCs to hypoxia could be manifested within minutes by the re-arrangements in the cytoskeletal protein network. The sensing mechanism of hypoxia by chemoreceptors in carotid bodies occurs rapidly in the blood (within seconds) (Prabhakar and Semenza, 2012). However, cellular responses to reduced pO_2_ depend on the duration of hypoxia; pre-existing proteins such as metabolic enzymes are activated within minutes but reprogramming of gene expression and subsequent physiological changes triggered by hypoxia requires several hours or days (Michiels, 2004; Prabhakar and Semenza, 2012, 2015). Hypoxia-induced improvements of RBC deformability may be revealed by long term deoxygenation where these improvements are no longer increased upon shear stress exposure.

**FIGURE 10:**
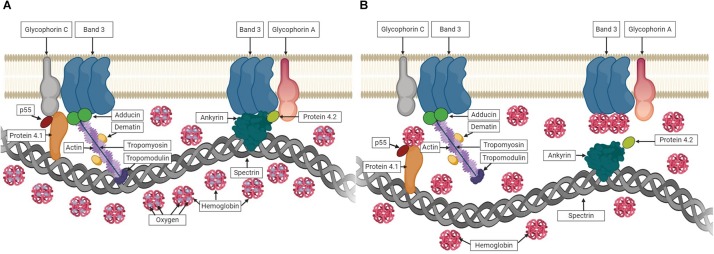
The associations between hemoglobin (Hb) and RBC membrane proteins. **(A)** shows Hb carrying oxygen and no interaction with membrane proteins in oxygenated state. **(B)** shows the association of Hb with membrane proteins while carrying no oxygen in deoxygenated state.

In healthy RBCs, conformational changes in hemoglobin (Hb) are determined by its oxygen saturation. In deoxygenated conditions, deoxyHb was shown to interact with Band 3 that causes the dissociation of Band 3-ankyrin bridge (Stefanovic et al., 2013; Chu et al., 2016). Similarly, deoxyHb can also interact with Protein 4.1 in deoxygenated state, which was shown by the rupture of Protein 4.1-Glycophorine C bridge upon deoxygenation (Chang and Low, 2001). These events under deoxygenated conditions could weaken the membrane-cytoskeletal associations and might explain the improved RBC deformability observed in the present study. The associations between Hb and RBC membrane proteins in oxygenated and deoxygenated states are illustrated in Figure 10. Furthermore, we measured the deformability of RBC ghosts to exclude the effect of Hb. However, no significant change was recorded between oxygenated and deoxygenated states (Supplementary Table 2). In addition, EI of ghosts at high SS levels (>5.15 Pa) decreased to negative values showing that RBC ghosts are unable to withstand high SS levels and subsequently begin to fragment. These results reveal that improvements in shear-induced responses of RBCs in deoxygenated conditions could be due to the associations between Hb and RBC membrane proteins.

Ion transporters (K-Cl, Na-K-2Cl cotransporters and Na/H exchanger) in RBC membrane are known to be O_2_-dependent and some ion channels and glucose transporters appear to be O_2_-sensitive (Gibson et al., 2000; Drew et al., 2004). It is postulated that oxygenation triggers swelling-activated ion transport (K-Cl cotransport), whereas deoxygenation stimulates shrinkage-activated transport (Na-K-2Cl cotransport and Na/H exchange) (Gibson et al., 2000; Barvitenko et al., 2005). Thus, it is likely that the volume of RBCs shows a decreasing trend in deoxygenation. However, reduced volume would increase the surface area to volume ratio when the surface area is constant, which would improve RBC deformability in deoxygenated conditions. Because higher deformability depends on high surface area to volume ratio (Cooke and Lim, 2007). This event would further support our findings in the present study. Nevertheless, specific organization of actin and spectrin in the cytoskeleton is necessary for the generation and transduction of signals triggered by cell volume alterations (Lauf and Theg, 1980; Pedersen et al., 2001). RBC membrane viscoelasticity with the skeletal protein network is considered as the most important contributor to deformability (Chien and Sung, 1990; Mohandas and Gallagher, 2008; Barvitenko et al., 2013). RBC membrane mechanical properties appear to have more significant roles than minimal volume changes for the maintenance of RBC deformability. Furthermore, divalent cations such as calcium (Ca^2+^) and Mg^2+^ are known to be involved in modulating some protein-protein interactions in RBC membrane. Free intracellular magnesium (Mg^2+^) concentration increases in deoxygenated conditions since DPG and ATP bind to deoxyHb and are not able to chelate Mg^2+^ ions (Bunn et al., 1971; Flatman, 1980). The binding of tropomyosin to F-actin is Mg^2+^ dependent (Fowler and Bennett, 1984). Erythrocyte membranes are shown to lack tropomyosin in the absence of Mg^2+^ and fragment rapidly when subjected to shear (An et al., 2007). Therefore, it is expected that increased binding of tropomyosin to actin in deoxygenated conditions restores the mechanical stability of erythrocyte membrane. Similarly, spectrin inter-dimer cross-linking is promoted by Mg^2+^ ions which shows spectrin-spectrin interactions are also Mg^2+^ dependent (Beaven et al., 1990). The discoid form of erythrocytes is stabilized by Mg^2+^ ions due to spectrin cross-linking, however, the elastic properties of the membrane is not grossly affected by intracellular Mg^2+^ (Beaven et al., 1990).

Cellular mechanisms involving nitric oxide (NO) and ATP release from RBCs are implemented for vasodilation in hypoxic conditions to enhance the blood flow and to supply O_2_ to tissues. Therefore, it is not surprising that RBCs could also increase their deformability in deoxygenated conditions. This would be particularly important for RBCs as they go through microcirculation in order to provide adequate O_2_ to tissues along with the effect of increased blood flow. Furthermore, a close relationship between ATP and RBC deformability was reported by many authors that a decrease in deformability attenuates ATP release (Sridharan et al., 2010; Prabhakar and Semenza, 2012; Thuet et al., 2012) whereas an increase enhances it (Raththagala et al., 2010; Forsyth et al., 2012; Thuet et al., 2012). A similar relationship with NO was also suggested in several studies referring to the reduction in extracellular NO decreased RBC deformability (Starzyk et al., 1997; Bor-Kucukatay et al., 2003). On the other hand, induction of NO synthesis or maintenance of NO bioavailability increased RBC deformability (Korbut and Gryglewski, 1993; Suhr et al., 2012; Grau et al., 2013). Instead of NO synthesis, RBCs favor the reduction of nitrite by deoxyHb to form NO in hypoxic conditions, so that RBC deformability is somehow guaranteed in severe hypoxia (Grau et al., 2016). Increased RBC deformability in hypoxic conditions in parallel with the release of ATP and NO suggests that RBC itself contributes to the hypoxic vasodilation in order to provide proper blood flow in microcirculation.

This is the first study that calculates EI distribution in oxygenated and deoxygenated conditions in a realistic 3D model. Still, there are several studies that model these parameters using one dimensional or lumped models in relatively simplified geometries. Imaging modalities, when combined with CFD, can provide information on the localized blood flow path, heat exchange, pressure drop, stress analysis, mass transfer of oxygen and carbon dioxide, difference convection-diffusion models, blood stagnation, thrombogenicity, etc. (D’Onofrio et al., 2017). Oxygenation has been investigated in computational (D’Onofrio et al., 2017; Lee et al., 2017) and experimental (Stobdan et al., 2015; Lv et al., 2018) simulation studies. D’Onofrio et al. (2017) have developed a 3D computational model of a blood oxygenator reconstructed from micro-CT scans and studied the localized functional behavior in terms of oxygenation. Their results suggest that, even with a significantly reduced the length of the oxygenator, sufficient oxygenation and a potential decrease of the thrombogenicity could be achieved. Another computational study investigates the factors that predispose the renal cortex to hypoxia (Lee et al., 2017). Simulation results suggest a possible pathway through which the capillary rarefaction might greatly increase the risk of renal hypoxia causing acute kidney injury. Lv et al. (2018) have designed a portable phantom device to simulate tissue oxygenation and blood perfusion experimentally. They have performed an initial evaluation of the feasibility of the integrated system, which combines the perfusion system and a 3D printed tissue-simulating phantom to simulate tissue oxygenation and blood perfusion. Their results indicate that the portable device has the potential to be a standardized device which can be used for assessing the performance of multispectral, laser spectral and other optical diagnostic imaging systems. Another experimental study analyses if endothelin receptor B improves cardiac tolerance to hypoxia in genetically engineered heterozygote mice (Stobdan et al., 2015). Vital organ hypoxic areas have been measured using immunohistochemistry staining for pimonidazole bound to hypoxic zones. All these studies suggest the advantage of simulation techniques for predicting oxygen level efficiently and accurately in blood and tissue without using human subjects.

The experimental results can determine the relationship between the stress and EI, however, this is limited with discrete values and the capacity of the test setup. The main contribution of the computational part of this study is the quantification and unveiling the distribution of the EI values of a physiological flow condition in a subject-specific artery geometry. Simulation results reveal that the highest EI is around the bifurcation region due to stagnation of the flow. Other high EI regions are around the walls except for the sinus diverging wall and mid sinus section. The high-stress regions tend to have higher EI values, but these regions are not obvious due to complex hemodynamic flow. The magnitude of the shear stress is determined by the multiplication of blood viscosity and velocity gradients. Velocity gradients are high around the walls due to no slip at the walls, but viscosity is higher at regions far from the wall, on the contrary, due to the shear thinning property (non-Newtonian flow) of the blood. Since the blood has shear thinning effect, especially at lower shear rates, a non-Newtonian model is used in the current simulation. Thus, the difference between the Newtonian and non-Newtonian model is higher at outer wall regions at daughter arteries (internal and external carotid arteries) as reported in the literature (Chen and Lu, 2004). Other profiles along the internal or external carotid artery shows more parabolic and symmetric velocity distributions as verified by the study (Chen and Lu, 2004).

The exact geometry used in this study or a very similar one has been used in several other studies (Perktold et al., 1991; Perktold and Rappitsch, 1995; Zhao et al., 2000; Sousa et al., 2011). Furthermore, there are many other studies which use a patient specific carotid artery geometry as we have done in the current study to analyze the hemodynamics in the vasculature (Piskin and Celebi, 2013; Dai et al., 2018; Xu et al., 2018). They used CFD simulation and medical imaging techniques to visualize velocity, pressure and wall shear distribution. So, we have compared our results to those studies to verify our findings in terms of hemodynamics. Figures 5B,C present the velocity contours and the streamlines at the bifurcation region of the main carotid artery into two daughter arteries. Figure 6 shows the velocity contour and vector distribution and velocity streamlines at the mid cross section throughout the whole domain. The low velocity and vorticity zone near the outer wall of the internal carotid artery are very similar to the reports of other simulation studies (Perktold et al., 1991; Sousa et al., 2011). During the acceleration phase of the cardiac cycle, the pressure distribution shows a linear gradual decrease through the main carotid artery inlet to the internal and external artery outlets as presented in Figure 7A. The same gradual behavior can be observed in other studies (Piskin and Celebi, 2013; Xu et al., 2018). Finally, the WSS distribution on the wall of the vasculature is given in Figure 7B. We can observe very similar patterns in the study (Dai et al., 2018; Xu et al., 2018): higher values near the stagnation point and inner walls of internal and external carotid arteries.

Simulation results revealed that the velocity gradient dominates the generation of the shear stress but the shear thinning effect of the blood has a minor effect on it. Although the performed simulation can be accepted as quite realistic, there are several limitations in the current study. The walls of the carotid artery have been modeled as rigid but actually, they are compliant. The geometry of the model is limited by the main carotid artery and its daughter arteries: internal and external carotid arteries. The domain can be extended to cover the smaller arteries and the capillaries although this will require much more computational source. However, the properties of the arterial flow and the capillary flow would not significantly differ from each other in terms of continuum mechanics modeling. Translational information obtained from arteries would be also applicable for the arterioles and the capillaries as long as the blood flow is continuous. Although numerical data could be changed depending on the size of the artery or the capillary and the blood flow rate, the principal of EI distribution throughout the blood vessel would be the same; as the highly elongated cells are concatenated close to the vessel wall due to the shear rate distribution throughout the lumen. Nevertheless, the physiological consequences of RBC deformability in large vessels are not straightforward. In this study we aim to model the blood flow simulation in a healthy artery to predict the distribution of the EI with regard to the shear stress. Since this kind of simulation is done the first time in the literature, to the best of our knowledge, we used a well-known and widely studied artery instead of small capillary arteries so that we would easily be able to verify our results. Also, the extent of elongation is related with the viscosity thus the non-Newtonian behavior of the blood flow. These effects are important in large vessels as well as smaller arteries since it will change the flow behavior and thus the stress distribution on the wall. Wall shear stress distribution is highly correlated with plaque formation and atherosclerosis (Dhawan et al., 2010). Furthermore, the distribution of the shear stress and thus EI are expected to be very similar in smaller arteries due to the physics behind the continuum fluid dynamics: Relatively higher shear stresses closer to the wall or stagnation (bifurcation) regions and lower shear stresses at the center of the artery.

## Conclusion

Red blood cells can adapt themselves to hypoxic conditions by improving their deformability under physiological shear forces; presumably to enhance blood flow and provide oxygen to tissues. This would support the notion that RBCs are not only oxygen carriers but also blood flow regulators by modulating their mechanical properties. Shear stress at physiological levels, that RBCs are continuously exposed to in the circulation, is a necessary precondition for RBCs to enable them to pass easily through capillaries in hypoxic conditions. Determination of RBC mechanical responses to hypoxia and the precise estimation of blood flow can be performed by computational tools which could allow us to design perfusion models of tissues that need or lack oxygen. This would be particularly important for monitoring of blood perfusion in cardiovascular or metabolic diseases such as diabetes mellitus where flow properties of blood are impaired, RBC deformability is reduced and oxygen transport is deteriorated. Besides, computational prediction of blood flow in hypoxic tumor tissues might provide useful information on the angiogenesis and the malignant progression of cancer. In addition, clinical effectiveness and duration of hyperbaric oxygen therapy could be evaluated by simulation-based hemodynamic investigation to assess oxygen conformance. As a future study, the disease-specific geometries of real patients can be studied for a better understanding of the effects of hypoxia on blood flow for the possible prognosis and treatment.

## Data Availability Statement

The raw data supporting the findings of this study will be made available by the authors, without undue reservation, to any qualified researcher.

## Ethics Statement

The studies involving human participants were reviewed and approved by the Ethics Committee of Koç University, School of Medicine (IRB:020/2012). The patients/participants provided their written informed consent to participate in this study.

## Author Contributions

EU and SP contributed equally to this study. EU, SP, AA, and AE conducted the experiments and performed the data analysis. OY supervised the experimental study design and interpretation. EU, SP, and OY wrote the manuscript. EU, SP, AA, AE, and OY read and approved the final version of the manuscript.

## Conflict of Interest

The authors declare that the research was conducted in the absence of any commercial or financial relationships that could be construed as a potential conflict of interest.

## Footnotes

1 https://grabcad.com/
