# Supplementary material for: From Experiments to Simulation: Shear-Induced Responses of Red Blood Cells to Different Oxygen Saturation Levels
Source: Front Physiol. 2020 Jan 22;10:1559. doi: 10.3389/fphys.2019.01559 (PMC6987081; doi:10.3389/fphys.2019.01559)
Supplement: Supplementary file 1 [file Data_Sheet_1.pdf]

Supplementary Material

A diligent mesh density sensitivity analysis was performed on the carotid artery geometry (**S1 Fig**) based on achieving a relative difference of less than 5% variations in the velocity (**S2 Fig**). The model geometry was discretized using curvature and radius dependent adaptive mesh elements. Grid sensitivity analysis was conducted using grids of decreasing mesh size (starting with 1.0 mm elements up to 0.25 mm) as presented in **S1 Table**. Regular mesh size (0.34 mm) was selected for meshing the whole domain and this mesh size was used in all simulations. An additional sensitivity analysis was conducted for time step size and the resulting size was found to be 0.01s as demonstrated in (**S2 Fig**). For a typical high-density spatial grid with a total of ~500k fluid elements and a grid spacing of 0.34 mm, a simulation time step size of 10^{-2} seconds in physical time is required to achieve the convergence. Simulations are continued until convergence of 10^{-5} residue. The conservation of mass was checked for verification of all cases. Total difference between inlet flow and outlet flow is less than 0.1 L/min (10-8 kg/s). Simulations were run for all configurations under the same inlet and outlet boundary conditions.

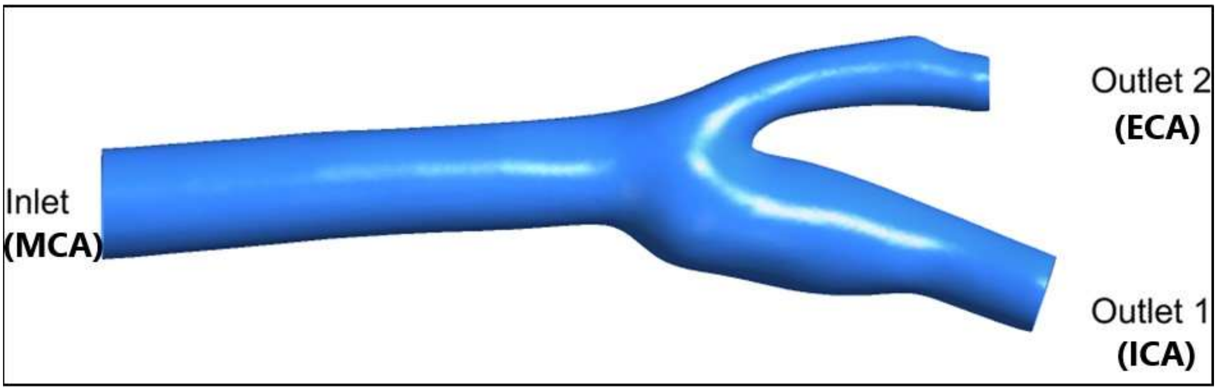

S1 Fig. Three-dimensional geometry of the carotid artery. Bifurcation model with one inlet and two outlets. MCA: Main Carotid Artery, ICA: Internal Carotid Artery, ECA: External Carotid Artery.

S1 Table. Mesh sensitivity table. The table presenting the mesh element size versus the number of elements.

Name of case	Element size (mm)	Number of elements
Coarser	1	75934
Coarse	0.5	212842
Regular	0.34	517258
Fine	0.25	1244777

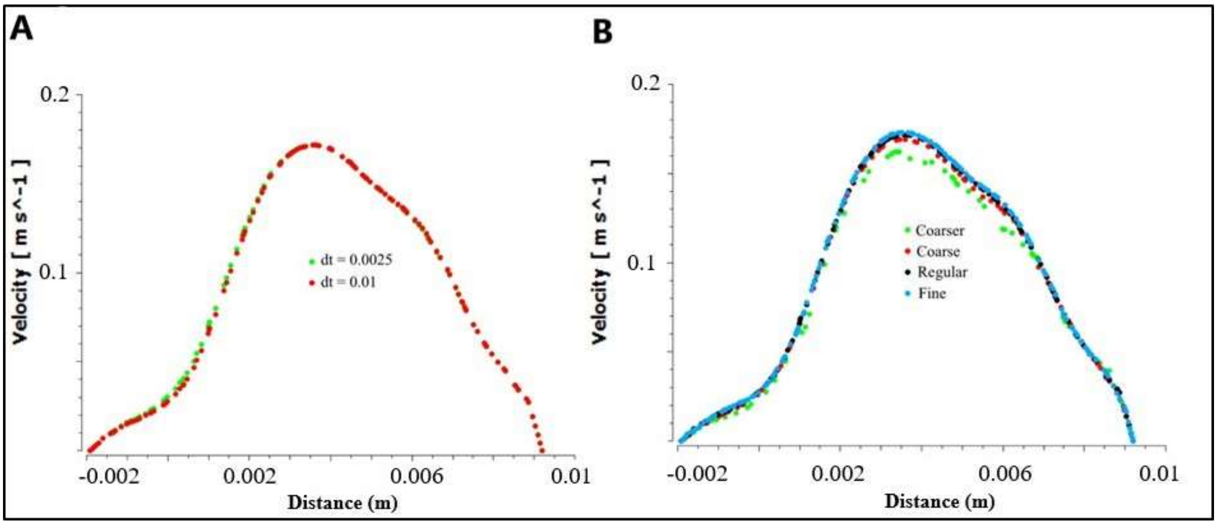

S2 Fig. Sensitivity analysis for simulation. (A) mesh and **(B)** time step size for sensitivity analysis. Mesh sizes are 0.25, 0.34. 0.5 and 1 mm for fine, regular, coarse and coarser mesh sizes. dt represents the time step size of the simulation. Step sizes of 0.0025 and 0.01 have been tested on the simulation.

S2 Table. Ghost elongation index values at low shear stress levels. The table presenting the shear stress versus the elongation index (EI) of ghosts (n=6) for oxygenated and deoxygenated states. Data is given as mean±standard deviation.

	**Before SS**		**After SS**	
Shear stress (Pa)	**Oxygenation**	**Deoxygenation**	**Oxygenation**	**Deoxygenation**
0.3	0.037±0.025	0.036±0.024	0.028±0.013	0.027±0.01
0.53	0.048±0.024	0.047±0.021	0.051±0.018	0.052±0.019
0.94	0.062±0.016	0.061±0.016	0.068±0.017	0.068±0.018
1.65	0.082±0.018	0.08±0.016	0.081±0.022	0.082±0.024
2.91	0.102±0.044	0.099±0.042	0.096±0.048	0.094±0.05
5.15	0.112±0.077	0.109±0.074	0.103±0.078	0.102±0.078

S3 Table. Elongation index values at each shear stress level. Table presenting the shear stress versus the elongation index (EI) of erythrocytes for oxygenated and deoxygenated blood samples. EI values are the average values of 10 different subjects from two replicative measurements.

Shear Stress (Pa)	EI for Oxygenated Blood	EI for Deoxygenated Blood
0,3	0,084	0,111
0,53	0,164	0,196
0,94	0,251	0,283
1,65	0,339	0,368
2,91	0,435	0,455
5,15	0,506	0,518
9,09	0,555	0,563
16,04	0,593	0,600
28,32	0,620	0,630
50	0,643	0,658
